# On the Prospects of In Situ Conservation of Medicinal- and Aromatic-Plant Genetic Resources at Ancient-Hillfort Sites: A Case Study from Lithuania

**DOI:** 10.3390/plants12040861

**Published:** 2023-02-14

**Authors:** Juozas Labokas, Birutė Karpavičienė

**Affiliations:** Laboratory of Economic Botany, Nature Research Centre, LT-08412 Vilnius, Lithuania

**Keywords:** archaeological site, cover-abundance of species, protected area, site evaluation, site-specific, species-specific, target species, threat assessment

## Abstract

Twenty-three ancient-hillfort sites were investigated to evaluate the potential for the in situ conservation of medicinal- and aromatic-plant populations. An evaluation of the site’s suitability was carried out by employing three major groups of criteria: species-specific, site-specific, and threat assessment. The species-specific criteria included the total species number, target species number, the cover-abundance of the target species estimated by mean Braun–Blanquet score, and, as an additional criterion, the number and cover-abundance of crop wild relatives. The site-specific criteria included site evaluation with respect to climatic region, the area size of a site, the habitat type, and the site’s protection status. The threat assessment was focused on anthropogenic activities, such as recreational, agricultural, and others. The total number of vascular plant species inventoried was 264, including 82 species of medicinal and aromatic plants (MAP). There was a strong and highly significant correlation between the total and the MAP species numbers (r_s_ = 0.77, *p* < 0.001), and the two most species-rich sites, Žuklijai and Pamiškė, contained the highest total and MAP species numbers. The investigated hillfort sites covered the populations of 49 species, or about 33% of the priority species list, with 5 or more populations. The most frequent species, *Hypericum perforatum*, occurred at 21 sites. The twenty-three hillfort sites represent three of the four climatic regions and six of the ten climatic subregions of Lithuania. Although these hillfort sites are quite small (1.24 ± 0.75 ha on average, without buffer zone), they are scattered across the country and are state-protected as archaeological objects, which makes them suitable for the in situ conservation of MAP genetic resources. In addition, seven hillfort sites (30.4% of the investigated ones) belong to the European network of special areas of conservation of habitats (Natura 2000), thus increasing their international importance. The threat assessment showed that anthropogenic activities (recreational, agricultural, etc.) are among the major factors affecting target-species populations.

## 1. Introduction

Medicinal and aromatic plants (MAPs) make one of the largest groups of plants consumed by people. Globally, between 50,000 and 80,000 flowering plant species are used for medicinal purposes and collected predominantly from wild habitats. In Europe, out of over 1300 medicinal plants used, 90% are harvested from wild resources [1]. The latest FAO report [2] revealed that the demand for wild-plant ingredients is growing rapidly, having grown by over 75% in value over the past two decades. 

Therefore, it is evident that the in situ conservation of MAPs should receive more focus than ever before. This is mostly important because of the role the intraspecific or intrapopulational diversity of species play in their adaptation to changing environments. Moore et al. [3] observed that plant-trait variance linked to both macro- and micro-environmental variation can also evolve and may respond even more strongly to selection than mean trait values. As global climate change alters the environments of plants, interactions between genes and the environment could determine the availability of the genetic variance needed to adapt to new environments.

To support in situ conservation, some tools have already been developed to help select target plant populations and to implement their management [4,5]. Nevertheless, site selection for the in situ conservation of MAP genetic resources remains a challenge as most of the target species are not legally protected and, thus, are often ignored by land users. Even in protected areas, various challenges are encountered regarding MAP conservation as these areas require dedicated management. Therefore, we focused on specific protected areas, i.e., archaeological sites, such as hillforts, which are managed to maintain grassland vegetation, with to the aim of selectively employing them as MAP genetic reserves. 

As stated by the typical protection regulations on immovable cultural values approved by the Government of Lithuania [6], archaeological sites include ancient production sites (workshops for tar-burning, charcoal burning, metal smelting), ancient mining sites (bog ore, amber and flint mines), ancient defensive installations (fortified ancient military camps, hillforts, castle sites, ancient defensive fortifications), ancient settlements (manors, fortified ancient settlements, villages, city sites, ancient settlements, homesteads), ancient hydrotechnical facilities (ancient canals, wells and ponds), ancient cult sites (ceremonial stones, paleo-astronomical facilities, temple and monastery sites), ancient burial sites (cemeteries, barrows, barrow complexes, cremation sites), ancient transport sites (ancient roads, wharves, bridges and ports), and ancient agricultural sites. In Lithuania, the most numerous of these are hillforts.

Hillforts are sites of ancient, fortified residences, single households, elite residences, whole villages, or even urban settlements built mostly on the tops of hills. Although the term primarily refers to those in Iron-Age Europe, similar structures are found throughout the world, spanning various time periods [7]. In Lithuania, as well as in the whole of Europe, fortified hills were used as social dwelling places from the beginning of the Late Bronze Age, and they were intensively used until the beginning of the 15th century [8]. The Baltic hillforts are outstanding natural-landscape formations: naturally formed hills and riverbanks with external earth fortifications encircling the hilltops [9]. Hillforts are the best known and the most beautiful archaeological monuments in Lithuania. Their total number is close to one thousand [10] and they are scattered all over the country. Currently, many hillforts are overgrown with forests, some have been transformed into recreational and festival sites, while others were badly damaged by ploughing and even levelled down [9]. Nevertheless, many of these archaeological sites have been regularly mowed for many years. 

Mowing prevents the development of land into scrub and woodland and allows the formation of semi-natural meadows, because the continued existence of many boreal grasslands depends on some form of human activity [11]. A long history of human land use had a strong influence on ecosystems and landscapes in the boreal forest region of Northern Europe and created semi-natural habitats of high conservation value [12]. As cultural objects of high ecological value, many hillforts could be favorable sites for the conservation of MAP as well as other economic plant-genetic resources, such as crop wild relatives (CWR), which contribute to the conservational value of the sites. In addition, a significant number of hillfort sites in Lithuania belong to the European ecological network Natura 2000, a network of special areas of conservation. Therefore, these sites are under EU protection law, indicating their international importance. 

Thus, the objective of this study was to evaluate the potential for the in situ conservation of MAP genetic resources in hillfort sites that are appropriately managed.

## 2. Results and Discussion

### 2.1. Site Evaluation by Species-Specific Criteria

Species inventories of vascular plants were carried out at 23 hillfort sites across Lithuania in 2020 through 2022. The total number of species amounted to 264, including 82 MAP species (hereafter referred to as target species). The major characteristics of the hillfort sites studied and the number of species inventoried are presented in Table 1.

The total number of species ranged from 18 at Kampiniai to 96 at Pamiškė hillfort, while that of the target species varied from 8 in both Kampiniai and Šimoniai to 39 in Žuklijai. There was a strong and statistically significant correlation between the numbers of total and MAP species (r_s_ = 0.77, *p* < 0.001), and the two most species-rich sites, Žuklijai and Pamiškė, contained the highest total and MAP species numbers (Table 1). These two sites also showed relatively high percentages of target species, with Žuklijai amounting to 42.9% and Pamiškė 37.5%. An estimated trade-off between the MAP species’ richness and their population sizes showed that these hillforts were among the best, as not only were they the richest in MAP species, but they also featured population sizes of at least several target species each. However, the correlations between the percentage of target species and the total species numbers were not statistically significant (r_s_ = −0.24, *p* = 0.260 and r_s_ = 0.26, *p* = 0.224, respectively).

Not only the number of MAP species, but also their cover and abundance are important in assessing the value of sites for in situ conservation, as the bare numbers of target species do not characterize the actual status of their populations or their probability of survival. A 5-point scale assessing the number of target species and the mean Braun–Blanquet score revealed that more than a half of the sites (fifteen) with the highest score of five points and five sites received four points, with the rest falling into the three-point (one) and two-point (two) groups (Table 2).

As an additional site-evaluation criterion, the occurrence and abundance of crop wild relatives (CWR), not included on the MAP list, was considered (Table 1 and Table 2). This criterion contributes to the conservational value of a site. As shown in Table 1 and Table 2, the site evaluation by the number and cover-abundance of CWR species was very close to that of the MAP species. It was established that the Spearman’s correlation between the site-evaluation scores based on the MAP and CWR species numbers and the cover-abundance was moderate: r_s_ = 0.49, *p* = 0.016. This suggests that sites rich in MAP species are often also rich in CWR species.

The analysis of the target-species occurrences across the sites showed that 33 target species (40.2%) did not meet the requirement of a minimum of five populations for safe long-term conservation in situ [13], while 21 target species occurred in 10 and more sites (Table 3), which met the stricter requirement for a minimum of 10 populations [14]. Considering the total number of 150 priority MAP species [15], the 23 hillfort sites covered the populations of 49 species, or about 33% of the priority-species list, more or less sufficiently, i.e., with five or more populations, and some efforts should be undertaken to investigate more hillforts for the remaining the target species. However, even significantly increasing the number of hillfort sites investigated may not cover all the priority MAP species, as the prevailing habitat types on hillforts are mostly those with dry and normal soil-moisture conditions.

The most frequent species was Hypericum perforatum, occurring at 21 sites (Table 3), and the rarest species occurring at a single site were Cynoglossum officinale, Dryopteris filix-mas, Euphrasia officinalis, Gentiana cruciata, Hypericum maculatum, Humulus lupulus, Oenothera biennis, Polygonatum odoratum, Potentilla anserina, Rubus nessensis, Sambucus nigra, Scrophularia nodosa, Tussilago farfara, and Rumex thyrsiflorus.

An important factor in plants’ genetic conservation is their reproduction strategies. Meloni et al. [16] found that clonality appears to positively affect the genetic diversity of *Ruta microcarpa* by increasing allelic diversity, polymorphism, and heterozygosity. Moreover, clonal propagation seems to be a more successful reproductive strategy in small, isolated populations subjected to environmental stress. In our study, most of the target species are perennial and can reproduce not only sexually, but also clonally; therefore, even small areas, such as the hillforts we studied, are suitable for the conservation of genetic diversity.

The impact of sexual compared with clonal reproduction on the effectiveness of hillfort sites for conserving genetic diversity should be seen in the context of the application of maintenance measures, principally mowing. Regarding species with prevailing sexual reproduction, the timing of mowing is the most important factor in maintaining their survival. If the mowing of such species takes place early in the season, i.e., before mature seeds are produced, they can disappear, as their main reproduction mode, sexual reproduction, cannot take place. The species that reproduce mostly clonally are less susceptible to the timing of mowing but also need regular rejuvenation by sexual reproduction to maintain their genetic diversity. From the pragmatic point of view, it is much easier to conduct mowing later for all species, as they often grow intermixed with each other.

Focusing on the impact of mowing as a selective factor, it should be taken into account that the grassland communities on hillforts develop as the result of regular agricultural activities, such as hay making and grazing; thus, the plant species that occur there are adapted to these activities. In the current study, in a few hillfort sites, where greater numbers of trees and shrubs were recently removed, some fragments of vegetation may inevitably change in terms of species composition. Further, the timing of mowing can predetermine how strongly it will act as a selective factor. In general, if mowing takes place in June, i.e., before the seeds of most species are produced, their genetic diversity may significantly decline over time. Therefore, it is recommended to carry out mowing later, e.g., not earlier than after mid-July. On the other hand, in some parts of hillfort sites, mowing can be applied earlier when undesirable species are to be eliminated. This partial site mowing can also be applied when patches of valuable forage plants are intended for use in hay production while leaving some fragments of the grassland for seed production and, later, mowing. In all cases, the cut grass must be removed from the site within two weeks.

### 2.2. Site Evaluation by Site-Specific Criteria

**Figure 1:**
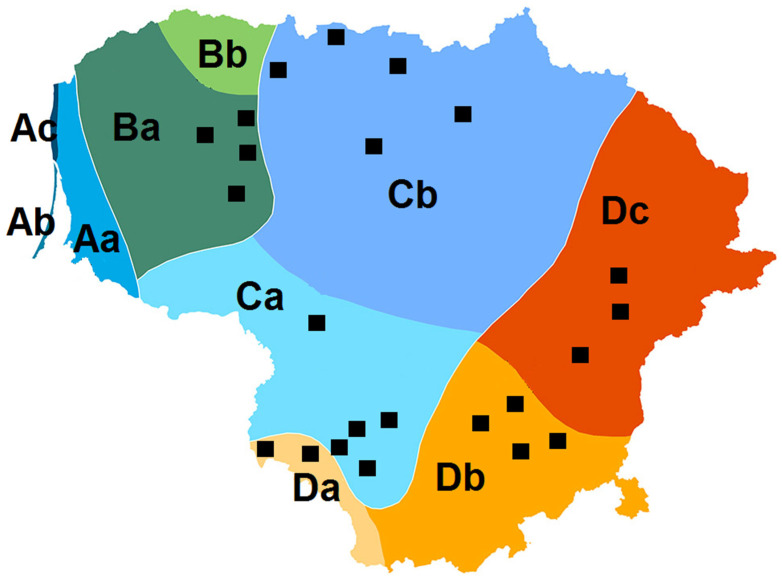
Distribution of hillfort sites (black squares) by climatic regions of Lithuania. Climatic regions: (**A**). Seaside (includes subregions Aa, Ab, and Ac); (**B**). Žemaičiai (includes subregions Ba and Bb); (**C**). Middle Lowland (includes subregions Ca and Cb); (**D**). South-Eastern Uplands (includes subregions Da, Db, and Dc). Each shade of color represents a different climatic subregion (map adapted from https://mapijoziai.lt/lietuvos-klimatas/, accessed on 2 January 2023).

These criteria may include site location with respect to the physical geographical region or climatic region, the area size of a site, the habitat type, and the site’s protection status. The twenty-three hillfort sites represent three of the four climatic regions and six of the ten climatic subregions of Lithuania (Figure 1). So far, only the seaside climatic region, which includes three subregions, does not feature a hillfort site. The same applies to one subregion in region B. Thus, further studies should focus on these unrepresented climatic subregions of the country. Furthermore, there is some underrepresentation of regions C and D, covering the largest parts of the country. Region C (the Middle Lowland) is distinguished by its absolute altitude of only 35–90 m. The total number of species in the investigated hillforts of regions C and D differed significantly (Kruskal–Wallis test, *p* = 0.032). The average number of species on the hillforts in the Middle Lowland was 49.1 ± 16.4 species, while in the region of the South-Eastern Uplands (region D) it was 69.8 ± 22.6 species. For all the other variables evaluated (the number and percentage of MAP and CWR species and site area), the mean values were also the lowest in region C, but the differences were not statistically significant. Scattered all over the country, hillforts cover all the country’s natural geomorphological and climatic subdivisions. This suggests that the target species found there are rich in ecogeographic diversity, which could be considered a proxy for genetic diversity [17,18].

Although the habitat types on hillfort sites are quite limited, as mentioned above, there may be some differences in soil fertility, slope aspects or microhabitat diversity between the sites, predetermining their species composition. As observed in southern Poland [19], the main factors predetermining the diversity of the flora of hillforts are the habitat characteristics and the method of management applied. Therefore, site evaluation by habitat type is highly relevant for determining hillfort-site suitability for the in situ conservation of MAP species. On the other hand, species diversity itself may be used as a proxy for habitat diversity, which was the case in the current study.

The site-area criterion is related to the well-known SLOSS (single large or several small) debate, to which Higgs and Usher [20] contributed significantly by arguing that a number of small reserves have more species than a single large reserve. Here, we suggest modifying this approach into SLOMS, i.e., single large or many small. This term applies to the findings in the current study, in which the hillfort -site evaluation by the site area inventoried showed that the sites varied from 0.22 ha (Šakališkiai) to 2.74 ha (Bendžiukai) (Table 1), with an average size of 1.24 ± 0.75 ha. Based on our previous study [4], a minimum site size of 0.4–0.5 ha could be applied to hillforts as well, but the neighborhood of a site should be considered in terms of whether it is favorable for the conservation of target-species populations (e.g., whether it is a protected area, a natural grassland, or an arable land). Only the percentage of target species correlated with site area moderately (r_s_ = 0.48, *p* = 0.020). The correlations between the total species and the target-species numbers and area were negligible (r_s_ = −0.07, *p* = 0.753 and r_s_ = 0.16, *p* = 0.453, respectively). Although the sites are quite small, as a kind of compensation for this, quite large buffer zones were established around most of the hillforts to ensure better protection of these archaeological objects. Thus, the total area of a state-protected hillfort site, which includes both a core and a buffer zone, is usually up to several times larger than that indicated in Table 1. In addition, all the hillfort sites were under state land ownership. Moreover, seven of the hillfort sites (30.4% of the investigated ones) belong to the Natura 2000 network of special areas of habitat conservation (Table 1), making them internationally important. Unambiguously, according to these criteria, all the investigated hillfort sites received the highest evaluation points, although a considerably higher number of sites should be investigated and included into the in situ conservation plan of MAP species, taking into account their small size. 

### 2.3. Site Evaluation by Threat Assessment

Further, we tried to evaluate each of the studied hillfort sites by threat assessment and overall suitability for long-term conservation as genetic reserves of MAP species. All the major factors affecting the hillfort sites are related to anthropogenic activities. These can be grouped as presented in Table 4, below. 

As shown in Table 4, all the investigated hillfort sites were affected by various kinds of anthropogenic activity or the abandonment of maintenance activities. Eleven sites were affected positively, mainly by grassland-maintenance methods, with estimated scores from one to four; twenty sites were estimated to have been affected negatively by such factors as the planting of cultivated species (Šimoniai), over-mowing (Pavištytis), trampling (Medvėgalis, Šatrija, Varnupiai), ploughing (Kampiniai), sand or gravel extraction (Bernotai), the introduction of invasive alien species (Moteraitis, Šakališkiai, and Žuklijai), and the abandonment of habitat-maintenance activities (Tričiai, Pamiškė, and Bendžiukai). Sometimes, similar activities can have the opposite effect. For example, shrub and tree removal had a positive effect in grassland communities and a negative effect in forest communities (Medvėgalis, Paplienija, Žemoji Panemunė). In some cases (Medvėgalis), the positive and negative effects of such actions were manifested in a single hillfort, on which both grassland and forest communities occurred. In addition, vegetation may be affected by different activities at the same site: the tops of the most frequently visited hillforts were trampled and over-mowed, while slopes are normally mown.

Although the flora of all the hillforts were dominated by native species, nineteen alien species were recorded, of which six occurred on more than one hillfort. The most common was *Lactuca serriola* L., which was found at seven sites in southern Lithuania. In addition to alien invasive species, *Calamagrostis epigejos* (L.) Roth, a native species, is becoming a major problem in unmown and abandoned grasslands. The spread and dominance of this species leads to the degradation of plant-species composition and the decline of species typical of mesic meadows, and an increased proportion of synanthropic species [21]. 

### 2.4. Other Considerations

A category of species called relics of cultivation was also observed at the hillfort sites. Considering the time of cultivation, Celka [22] divided the relics of cultivation into three groups: (i) relics of medieval cultivation (cultivated before the late 15th century); (ii) relics of cultivation in the modern era (cultivated since the 16th century); and (iii) relics of cultivation in both the Middle Ages and the modern era. However, in our study, these species were not found more frequently than in similar non-historic habitats: relics of medieval cultivation—*Malva alcea* (one site); relics of medieval-modern cultivation—*Artemisia absinthium* (two sites), *Origanum vulgare* (five sites); and *Pastinaca sativa* (one site). However, *Allium oleraceum*, which was used as a food, a spice, and a medicinal and cult plant during the first millennium A.D. and the medieval period in the Nordic countries [23], was found much more often at the hillfort sites we studied (13 sites) than in similar non-historic habitats. In Finland, *Allium oleraceum* is often found on Iron-Age mounds, in ancient-hilltop fortresses and in areas of medieval settlement, e.g., in connection with medieval castles and churches [23,24]. This suggests that some of the future research projects at archaeological sites might focus on relics of cultivation, which are currently underexplored in Lithuania. 

As observed by Celka et al. [25], the exceptional components of the cultural landscape, including archaeological sites such as hillforts, serve as habitat islands or, as we would suggest, refuge sites for various useful and interesting plant species. Thus, the role of hillforts, including, in some cases, their surroundings (e.g., when a hillfort is located in a larger protected area, such as a regional or national park) is important in the overall context of species-diversity conservation. Hillfort sites that are state-protected objects and cover relatively small areas are much more easily manageable for the conservation not only of populations of common medicinal- and aromatic-plant species, but also of rare and endangered species, along with historic heritage. Corresponding conservation plans and their implementation, particularly when dealing with different categories of species, depend firstly on the data available for the site of interest. Therefore, the regular observation of visitors’ impact and target-species monitoring are needed to achieve the best results with hillforts or similar archaeological sites, such as burial mounds or historic cemeteries. It is important to note that typical Lithuanian protection regulations for archaeological sites [6] allow such grassland-maintenance activities as mowing hay, cutting trees and bushes, and the grazing of small cattle (goats, sheep) in intact parts of these sites, and do not allow forest planting.

One may consider that the potentially effective in situ conservation of MAP species at hillfort sites, such as refuge habitats, might also be used for plant translocations as a further effective conservation tool for medicinal plants. This could be particularly relevant to the conservation of threatened species, such as when these are subjected to certain human activities, including the construction of roads or other vital communication lines. However, to apply this approach on a wider scale, specific research, including some experimental work, is needed. One of the major factors to be considered in the case of plant translocation is the environmental compatibility of populations in terms of the climatic subregions and ecogeographic conditions of habitats, including changes in plant communities. In any case, the protection regulations of archaeological sites [6], which generally do not allow digging of soil in hillfort sites, should be considered. In addition, as many hillfort sites belong to special areas of conservation, any efforts related to plant translocation should be undertaken by relevant experts. 

## 3. Conclusions

Most of the investigated hillfort sites are suitable for the long-term in situ conservation of medicinal- and aromatic-plant genetic resources. These species are often accompanied by crop wild relatives, which increase the conservational value of their sites. Additionally, some relics of cultivation were also found at the hillfort sites, indicating that these archaeological sites can provide several benefits in the field of biodiversity research and conservation alone. A critical factor enabling the in situ conservation of target species at hillfort sites is the persistent maintenance of open habitats through the mowing of sites and the control of their overgrowth by woody species. Usually, this maintenance is implemented under the supervision of protected area managers, in accordance with approved nature-management plans. Although these management plans are mostly intended to maintain an appropriate environment for protected archaeological objects (e.g., to reveal them and make them more attractive for visitors), they help to achieve the dual goal of conserving biodiversity and promoting historical heritage, contributing at the same time to the sustainability of archaeological sites. Considering the small average size of archaeological sites, a larger network of these sites should be created to ensure that a minimum of five populations of each target species are covered in all the climatic subregions of the country. Management plans for target-species populations should be developed to ensure the integrated in situ conservation of biodiversity at archaeological sites. Special areas of conservation for habitats should be of a particular interest in future research endeavors.

## 4. Materials and Methods

The study sites were selected on the basis of data from the Atlas of Mounds (https://www.piliakalniai.lt/, accessed on 1 February 2023). The cover-abundance of each plant species in the plant communities was evaluated according to the six-grade Braun–Blanquet scale, where the r score was not used and + score was substituted by 0.5 for the convenience of calculation of mean Braun–Blanquet scores. The taxonomy of species follows the accepted names of vascular plants of the European and Mediterranean vascular plant taxa provided by The World Flora Online [26]. The target MAP species were selected as in Labokas and Karpavičienė’s study [15] and CWR species as in Labokas et al.’s study [27]. As bare numbers of species do not reveal the actual quantity of plants, the mean Braun–Blanquet scores of MAP and CWR species were calculated to evaluate their cover-abundance. A five-point scale was used to evaluate the studied sites according to MAP- and CWR-species number and their cover-abundance as described in Labokas and Karpavičienė’s study [4], with ‘5’ representing the highest quality or state and ‘1’ representing the lowest quality of a site (Table 5). 

Site areas were estimated by using QGIS (http://www.qgis.org, accessed on 1 February 2023) and an online map at https://www.geoportal.lt/map/ (accessed on 1 February 2023).

Regarding site-specific criteria, hillfort sites were estimated with respect to the climatic subdivisions of the country and state land ownership, with both sets of data provided by the National Land Service under the Ministry of Agriculture (https://www.geoportal.lt/map#, accessed on 1 February 2023).

Occurrence of hillfort sites within the network of special areas of conservation (Natura 2000) was determined by using the cadastral maps of the State Service for Protected Areas [28].

For threat assessment, the baseline data used were from the reference list of threats and pressures maintained by the European Environment Agency at EIONET Central Data Repository [29].

The Shapiro–Wilk W test was used to test the normality of the distributions of data. Because the data did not match a normal distribution, non-parametric statistical methods were used. The relationship between variables was analyzed using Spearman’s rank correlation and differences between climatic regions were analyzed using the Kruskal–Wallis test. All statistical analyses were conducted using Statistica 10.0 (StatSoft) software package.

## Figures and Tables

**Table 1:** Major characteristics of hillfort sites studied and number of species inventoried.

Hillfort Name	Coordinates WGS-84	Year of Inventory	Total Species Number	MAP Species Number	MAP Species %	CWR * Species Number	Site-Area Inventoried, ha	Predominant Habitat Type	Special Area of Conservation
Vorėnai	55.35748, 25.60970	2022	78	20	25.6	13	0.50	Grassland	No
Bernotai	54.90947, 25.32245	2022	75	14	18.7	17	0.60	Grassland	No
Medvėgalis	55.62770, 22.39278	2020	86	27	31.4	11	2.05	Grassland	Yes
Rekčiai	56.07736, 22.85350	2020	68	24	35.3	15	1.04	Grassland	Yes
Šatrija	55.87270, 22.55800	2020	52	17	32.7	16	0.33	Grassland	Yes
Paplienija	55.83674, 22.17979	2020	56	22	39.3	15	0.81	Forest/Grassland	No
Žagarė	56.35472, 23.22667	2020	50	18	36.0	9	0.30	Grassland	Yes
Moteraitis	55.76457, 22.49970	2020	51	22	43.1	11	2.50	Grassland	Yes
Pavištytis	54.41533, 22.78184	2021	63	24	38.1	12	1.80	Grassland	Yes
Šakališkiai	54.55398, 23.68895	2021	71	26	36.6	8	0.22	Grassland	No
Varnupiai	54.48993, 23.53442	2021	38	16	42.1	9	0.50	Grassland	No
Padovinys	54.50951, 23.44372	2021	50	18	36.0	10	0.51	Grassland	No
Liudvinavas	54.47420, 23.34622	2021	51	19	37.3	12	1.80	Grassland	No
Kampiniai	54.44696, 23.19809	2021	18	8	44.4	5	1.90	Grassland	No
Žemoji Panemunė	55.05275, 23.45857	2021	21	11	52.4	2	1.90	Woodland/Grassland	No
Raginėnai	55.79289, 23.86468	2022	62	19	30.6	7	1.23	Grassland	Yes
Tričiai	56.10793, 24.02301	2022	53	16	30.2	14	0.39	Woodland/Grassland	No
Šimoniai	56.05512, 24.31670	2022	27	8	29.6	8	1.88	Woodland/Grassland	No
Žuklijai	54.50982, 24.68262	2022	91	39	42.9	13	1.31	Grassland	No
Einoronys	54.44435, 24.39077	2022	76	28	36.8	11	1.34	Grassland	No
Geruliai	54.53128, 24.27053	2022	71	30	42.3	11	1.11	Grassland	No
Pamiškė	54.62611, 24.51146	2022	96	36	37.5	22	1.74	Grassland	No
Bendžiukai	55.09028, 25.58466	2022	60	30	50.0	8	2.74	Grassland	No

* CWR—crop wild relative.

**Table 2:** Hillfort-site evaluation by MAP- and CWR-species cover-abundance estimated by mean Braun–Blanquet score.

Hillfort Name	Braun–Blanquet Score of MAP Species		Site Evaluation Score by MAP **	Braun–Blanquet Score for CWR Species		Site-Evaluation Score by CWR **
	Mean	SD *		Mean	SD	
Vorėnai	0.82	0.51	5	1.12	0.65	5
Bernotai	0.68	0.46	5	1.03	0.76	5
Medvėgalis	0.72	0.35	5	0.86	0.84	5
Rekčiai	0.87	0.64	5	0.93	0.50	5
Šatrija	1.00	1.06	4	1.13	0.89	5
Paplienija	0.95	0.69	4	0.90	0.63	5
Žagarė	1.03	0.79	5	1.11	0.96	4
Moteraitis	0.94	0.60	5	1.23	0.52	5
Pavištytis	0.79	0.49	5	0.88	0.43	5
Šakališkiai	0.86	0.59	5	0.81	0.53	4
Varnupiai	1.04	0.80	4	1.11	0.86	4
Padovinys	1.09	0.76	5	1.15	0.88	5
Liudvinavas	0.97	0.70	4	0.96	0.78	5
Kampiniai	0.88	0.52	2	1.50	0.71	3
Žemoji Panemunė	0.82	0.46	3	1.25	1.06	2
Raginėnai	0.68	0.37	4	1.43	0.53	4
Tričiai	0.97	0.72	4	0.86	0.53	5
Šimoniai	0.79	0.57	2	0.56	0.18	4
Žuklijai	0.76	0.43	5	0.92	0.67	5
Einoronys	0.73	0.46	5	1.45	0.99	5
Geruliai	0.75	0.55	5	0.86	0.45	5
Pamiškė	0.82	0.58	5	0.95	0.71	5
Bendžiukai	0.69	0.41	5	1.19	0.88	4

* SD—standard deviation. ** According to Labokas and Karpavičienė [4].

**Table 3:** Frequency of target-species occurrences across hillfort sites.

Species Name	Number of Sites Where the Species Occur
*Hypericum perforatum*	21
*Achillea millefolium*, *Agrimonia eupatoria*, *Thymus pulegioides*, *Solidago virgaurea*, *Plantago media*, *Quercus robur*	15–19
*Artemisia vulgaris*, *Pimpinella saxifraga*, *Allium oleraceum*, *Fragaria viridis*, *Urtica dioica*, *Equisetum arvense*, *Pinus sylvestris*, *Fragaria vesca*, *Pilosella officinarum*, *Plantago lanceolata*, *Primula veris*, *Rumex acetosa*, *Trifolium pratense*, *Sorbus aucuparia*	10–14
*Frangula alnus*, *Taraxacum officinale*, *Trifolium repens*, *Elymus repens*, *Prunus padus*, *Rhamnus cathartica*, *Crataegus monogyna*, *Picea abies*, *Betula pendula*, *Cichorium intybus*, *Fraxinus excelsior*, *Linaria vulgaris*, *Rosa canina*, *Rubus idaeus*, *Valeriana officinalis*, *Anchusa officinalis*, *Anthoxanthum odoratum*, *Carex arenaria*, *Corylus avellana*, *Geum urbanum*, *Helichrysum arenarium*, *Malus domestica*, *Malus sylvestris*, *Origanum vulgare*, *Pimpinella major*, *Populus tremula*, *Sedum acre*, *Tilia cordata*	5–9
*Alchemilla vulgaris*, *Glechoma hederacea*, *Viburnum opulus*, *Alnus incana*, *Epilobium angustifolium*, *Pulsatilla pratensis*, *Rubus caesius*, *Thymus oblongifolius*, *Verbascum thapsus*, *Artemisia absinthium*, *Asarum europaeum*, *Chelidonium majus*, *Convallaria majalis*, *Hepatica nobilis*, *Juniperus communis*, *Prunella vulgaris*, *Rumex crispus*, *Thymus serpyllum*, *Bistorta officinalis*, *Cynoglossum officinale*, *Dryopteris filix-mas*, *Euphrasia officinalis*, *Gentiana cruciata*, *Hypericum maculatum*, *Humulus lupulus*, *Oenothera biennis*, *Polygonatum odoratum*, *Potentilla anserina*, *Rubus nessensis*, *Sambucus nigra*, *Scrophularia nodosa*, *Tussilago farfara*, *Rumex thyrsiflorus*	1–4

**Table 4:** Evaluation of anthropogenic activities affecting target-species populations at hillfort sites observed.

Aim and Effect of Activity	Description of Activity	Hillfort Sites Concerned	Estimated Degree of Effect (1–Very Low, …, 5–Very High)
Recreational, positive	Regular mowing and woody-plant control	Medvėgalis, Šatrija, Raginėnai, Žagarė, Žuklijai	4
		Rekčiai, Geruliai	3
Recreational, negative	Planting of cultivated grasses and legumes, establishment of lawns, alleys	Šimoniai	4
	Over-mowing	Pavištytis	3
	Trampling	Žemoji Panemunė	1
Agricultural, positive	Regular haymaking/livestock grazing	Einoronys, Liudvinavas	1
		Padovinys, Vorėnai	3
		Varnupiai	2
Agricultural, negative	Ploughing, leveling up	Kampiniai	2
Mining, negative	Sand/gravel extraction	Bernotai	1
Other, negative	Introduction of invasive non-native species	Žuklijai, Šakališkiai	1
		Moteraitis	2
No activity, negative	Abandonment of grassland-maintenance activities	Tričiai, Pamiškė, Bendžiukai	2
	Abandonment of forest-habitat-maintenance activities	Paplienija	2

**Table 5:** Genetic site evaluation by number of target species and their cover-abundance (adapted from [4]).

Number of Target Species	Mean Braun–Blanquet Score *	Evaluation Score
1–5	0.5–1.0	1
	1.1–2.0	2
	2.1–3.0	3
	3.1–4.0	4
	4.1–5.0	5
6–10	0.5–1.0	2
	1.1–2.0	3
	2.1–3.0	4
	3.1–4.0	5
11–15	0.5–1.0	3
	1.1–2.0	4
	2.1–3.0	5
16–20	0.5–1.0	4
	1.1–2.0	5
>20	0.5–1.0	5

* Braun–Blanquet scores for species cover-abundance: ‘+’ denotes cover of less than 1%, with very few individuals (here we treat it as ‘0.5’); ‘1’ denotes 1–5% cover and many individuals; ‘2’—cover 5–25%; ‘3’—cover 25–50%; ‘4’—cover 50–75%; ‘5’—cover 75–100%.

## Data Availability

All data generated during this study are included in this article.
